# A Manual of Differential Medical Diagnosis

**Published:** 1886-12

**Authors:** 


1. MANUAL OF DIFFERENTIAL MEDICAL DIAGNOSIS. *By* CONDICT W. CUTLER, M.S., M.D. *12mo, pp. 161. New York:* G. P. PUTNAM'S SONS. *Chicago:* A. C. MCCLURG & COMPANY. *1886.*

